# The time is now: Student‐driven implementation of social justice and anti‐racism focused curricula in medical scientist training program education

**DOI:** 10.1096/fba.2020-00112

**Published:** 2021-03-16

**Authors:** Mackenzie Lemieux, Sneha Chaturvedi, Elizabeth Juarez Diaz, Lilianne Barbar, Maggie Bui, Danielle Isakov, Evan Lee, Paul Lee, Blake Sells, Tiankai Yin

**Affiliations:** ^1^ Washington University School of Medicine St, Louis MO USA

**Keywords:** anti‐racism, Black, COVID‐19, indigenous, MD‐PhD, People of Color (BIPOC), social justice

## Abstract

There exists a dearth of supplementary programs to educate physician–scientist trainees on anti‐racism and topics surrounding social justice in medicine and science. Education on these topics is critical to prevent the perpetuation of systemic racism within the institutions of academia and medicine. Students in the Washington University School of Medicine Medical Scientist Training Program and the Tri‐Institutional MD‐PhD Program developed journal clubs with curricula focused on social justice and anti‐racism for the summer of 2020. In this article, we describe the impact of the Washington University journal club on the education of first year MD‐PhD students and summarize the progress to date. The role of the journal club in the midst of the “double pandemic” of COVID‐19 and generational systemic racism is discussed, highlighting the need for such supplemental curricula in MD‐PhD programs nation‐wide.

## 1 IMPORTANCE AND VISION

In a year marked by the COVID‐19 pandemic and an uprising against racial injustice, the importance and urgency of educating ourselves on racism and how to be anti‐racist has never been more apparent. As we embark on our physician‐scientist training in the community of St. Louis, and in the United States, where science and medicine have histories steeped in injustices towards communities of color, we sought to create a platform to educate ourselves in order to uplift these communities in our careers and personal lives. Recently, societal inequalities have been exacerbated by the COVID‐19 pandemic, with marginalized communities being disproportionately affected.^1^, ^2^ While all of our lives have been impacted in some way by the global COVID‐19 pandemic, communities of color are simultaneously suffering from the ongoing pandemic of systemic racism.

As physician‐scientists in training, we have a fundamental responsibility to society to understand and respond appropriately to this “double pandemic.” Inspired by like‐minded students at the Weill Cornell Medical College, The Rockefeller University, and Memorial Sloan Kettering Tri‐Institutional (Tri‐I) MD‐PhD Program, we decided that creating a journal club to educate each other and discuss important issues regarding social justice and anti‐racism was a first step in our life‐long learning of these topics. In creating this journal club, we aimed to garner the knowledge necessary to understand historical traumas suffered by communities of color in the institutions of science and medicine. We felt the need to gain a deeper understanding of the inherent mistrust of the medical community that exists and how to dismantle it. These topics were addressed in the context of the social and structural determinants of health that already plague the health outcomes of populations of color. At the same time, this journal club served as a space for us to challenge each other to think about how to use our multifaceted roles as medical students and scientists, to address the issues that have led to poorer health outcomes for communities of color.

Currently, there is an urgency to put an end to the COVID‐19 pandemic, but the pandemic of systemic racism has plagued this nation since its conception and has taken countless lives and oppressed countless others in the process. Therefore, it is just as urgent for all of us to work together to end systemic racism. As future physician‐scientists, this journal club provides a critical component of our training to best advocate for our patients and continue to push the medical and scientific establishments to fight towards racial equity, instead of being complicit in a system that has taken so many lives.

## 2 BEGINNINGS IN NEW YORK CITY

In July of 2020, the onslaught of institutional violence against marginalized communities in the United States prompted two of the largest MD‐PhD programs in the country, the Tri‐I MD‐PhD Program and the Washington University School of Medicine (WUSM) Medical Scientist Training Program (MSTP), to take action. The entering Tri‐I MD‐PhD class was concerned that the formal education on social determinants of health through the medical school was not extensive, rigorous, or frequent enough to explicitly focus on how science and medicine contribute to structural racism. With the support of the Tri‐I leadership, they created an intersectional anti‐racist journal club to learn how to recognize and begin to address their own internalized racism and biases, identify how modern‐day institutions continue to enforce racial oppression, and discuss concrete solutions and actionable items to implement. The works featured in the syllabus were based on resources that academics, historians, activists, artists, scientists, and authors (many of whom self‐identify as Black, Indigenous, or People of Color) have put together over the years to learn, reflect, and shape behaviors that actively oppose racism and injustice.

## 3 FROM NEW YORK TO ST. LOUIS

The incoming MSTP students at WUSM in St. Louis became aware of the journal club started by the entering Tri‐I MD‐PhD class. Not only were we, as the incoming MSTP class, inspired to start our own journal club focused on these themes, but we were cognizant of the need to establish educational opportunities like this for MD‐PhD programs across the country. Since we had not officially started our formal MD‐PhD training, this journal club represented a timely opportunity to positively shape our class culture. The journal club was designed to serve as a safe environment to discuss social injustices in science and medicine within our MD‐PhD community. By sculpting the shared goals and values of our class around addressing bias, discrimination, and racial injustice, we hoped to go beyond enhancing our individual knowledge and influence positive change at a structural level.

## 4 EDUCATIONAL GOALS

The two main educational goals of the journal club were to increase our group awareness of the systemic racism that exists in the fields of medicine and academia, and to learn how to take specific and longitudinal actions to combat oppression and discrimination in our communities. Because of WUSM’s location in St. Louis, MO, specific consideration was given to the history of St. Louis to educate incoming students on the community they would be engaging with. Both scientific research and medical practice have long histories of exploiting marginalized communities in the United States, especially Black and Indigenous populations.^3^, ^4^, ^5^ This has caused generations of mistrust between affected communities and the healthcare system.

For decades, medical school students and administrators have advocated for a specific curriculum centered around social justice.^6^, ^7^, ^8^ Schools that have implemented social justice curricula have highlighted the importance of teaching students about these topics and shown that dedicated curricula can yield strategies to advance the conversation surrounding racism in healthcare.^9^, ^10^, ^11^ In addition, many medical schools have released commitments to combat institutional racism in other ways beyond the curriculum, including community partnerships and creating inclusive environments for a diverse workforce.^12^ Scientific journals have expressed similar commitments.^13^, ^14^

Unfortunately, patients, trainees, scientists, and physicians from marginalized groups continue to face injustice.^15^, ^16^, ^17^ The structural inequities caused by intersections between identity and health are exacerbated by the current COVID‐19 pandemic, where Black and Hispanic populations are disproportionately affected.^18^, ^19^ The impact of COVID‐19 on systemic racism has shown what many activists and experts have been saying for years: racism is a public health crisis.^2^, ^20^ However, unlike COVID‐19, systemic racism has been perpetuating inequities in healthcare for generations. Therefore, all healthcare workers, including physician‐scientists, need to be actively learning and implementing anti‐racist and trauma‐informed practices in the pursuit of health equity for all of our patients.

As future physician‐scientists, this journal club provides a critical component of our training to learn how to best advocate for our patients and continue to push the medical and scientific establishment to fight towards racial equity. As MD‐PhD students, we function at the intersection of the medical field and scientific academia. In our unique and privileged position, we not only have an increased obligation to educate ourselves on the historical and current forms of institutional racism that persist in this country, but are dually empowered to effect change throughout our training and our future careers.

## 5 INFRASTRUCTURE BUILD

To design the structure of the journal club, all interested students communicated over email and attended two preliminary Zoom meetings. The purpose of these meetings was to brainstorm a syllabus, journal club structure, and community guidelines to be followed in each session. In designing our journal club leadership format, we decided to use an empowered leadership approach.^21^ This leadership style distributes leadership and gives decision‐making power and autonomy to members of the journal club, minimizing the power held by a central leader.^21^ We ensured that all members of the journal club were integrally involved in creating the community guidelines and designing the structure of the curriculum. Involving all journal club participants in the initial planning allowed us to reach shared goals and values. All participants also had a chance to educate the group on a specific topic surrounding social justice in science and medicine. Additionally, this ensured that the topics covered were of primary interest to all participants.

**FIGURE 1:**
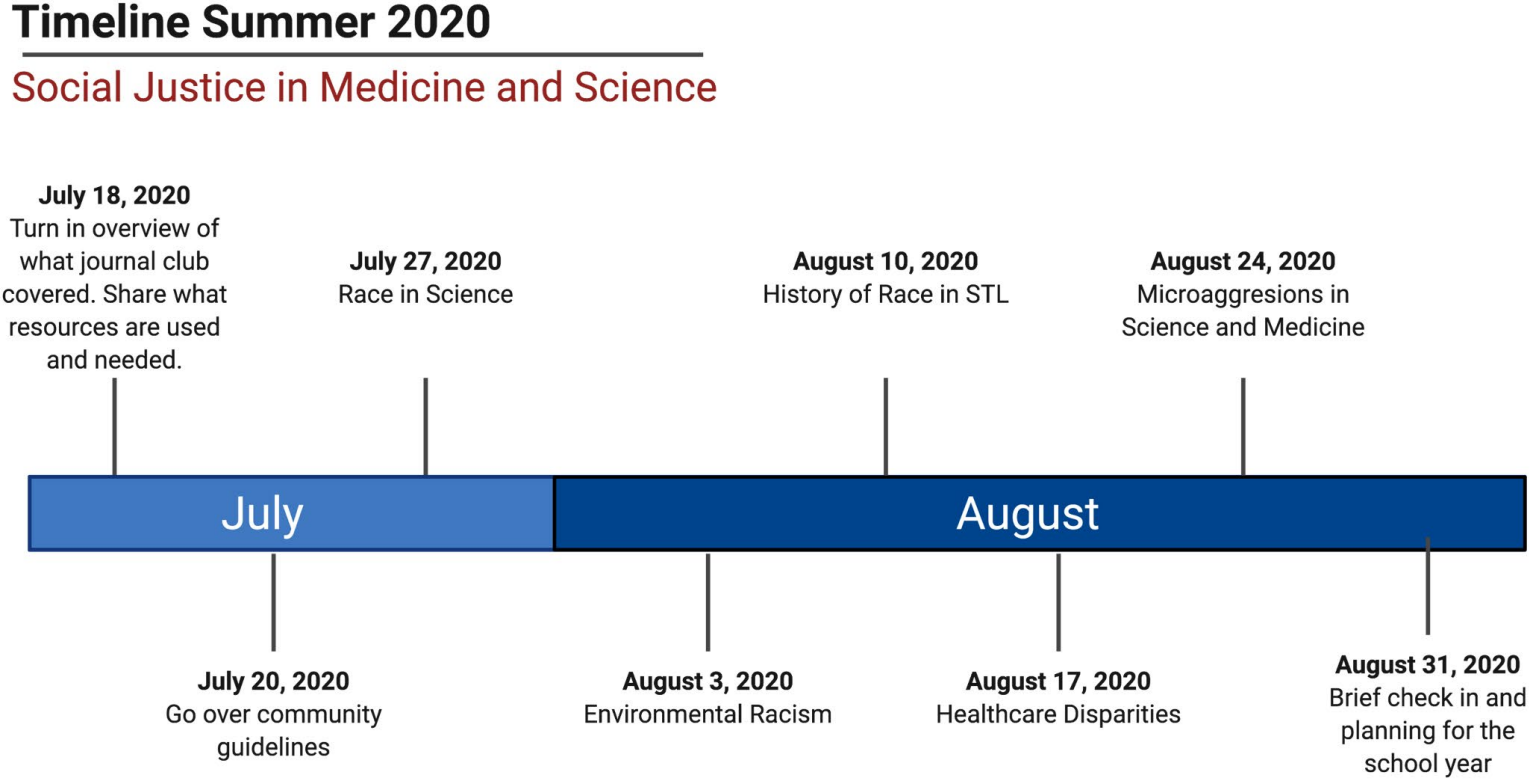
Social Justice in Science and Medicine Journal Club Timeline. Schematic of the structure used to implement the journal club throughout the summer of 2020. Created with BioRender.com

In the preliminary meetings, we brainstormed the topics that we felt were critical to address in a curriculum focused on the injustices present in both science and medicine. A survey was completed by the participants and used to assess which topics were perceived as most important to cover during the five journal club discussions to be held over the summer. The top five most voted topics were: the history of race in St. Louis, environmental racism, race in science, health disparities and cost, and microaggressions in science and medicine (Figure 1). The other topics were suggested included: health disparities and cost of care, racial and ethnic imbalances in academic citations, cultural humility, intersectionality of race and gender, Queer and POC rights, racial equity in biomedical science, and immigration in America. These topics that were not explicitly discussed in the summer, but were added to a list of topics to discuss throughout the year. For each journal club, groups of two or three students signed up to create a presentation, as well as facilitate and lead discussions around their preferred topic (Table 1). Each presenting group communicated over email and Zoom. A second preliminary survey was sent to the class prior to the first topic‐based discussion to assess class familiarity with topics to be covered and current levels of comfort discussing race in medicine and science in order to compare pre‐ and post‐perceived learning after the summer journal club.

**TABLE 1:** Journal Club Topics and Associated Resources. Table shows the list of required resources for each journal club session. Resources were chosen by student presenters and distributed via email. Additional resources were provided at the end of each presentation, which are not listed in the table

Meeting topic	List of resources
Race in Science	Racism and Research (Tuskegee Syphilis Study) “The Immortal Life of Henrietta Lacks” by Rebecca Skloot (Prologue only) Bidil—Assessing a Race‐Based Pharmaceutical (Annals of Family Medicine) “Science has a Racism Problem”(Cell 2020)
Environmental Racism	Robert Bullard: How Environmental Racism Shapes the US (video) 17:30 minutes long Toxic Cities: Neoliberalism and Environmental Racism in Flint and Detroit, Michigan (Sociology article) 11 pages, not including citations Environmental Racism Has Left Black Communities Especially Vulnerable to COVID‐19 (commentary article) ~5–10 min read Environmental Racism in St. Louis (Source article) 5‐minute summary required, 22‐page report optional (but highly encouraged!)
History of Race in St. Louis	Dred Scott v Sanford Supreme Court Decision (Introduction required) Mapping Decline: St. Louis and the American City Ferguson Activists Return To The Streets After Police Kill Another Black Man (Audio required) Mackenback 2009 ‐ Politics is nothing but medicine at a larger scale: Reflections on public health's biggest idea Pruitt‐Igoe: the troubled high‐rise that came to define urban America‐(Guardian article)
Healthcare Disparities	Medical Apartheid by Harriet Washington, Chapter 12 Disparities in Health and Health Care: Five Key Questions and Answers Racism and discrimination in health care: Providers and patients White & Stubblefield‐Tave 2016 Some Advice for Physicians and Other Clinicians Treating Minorities, Women, and Other Patients at Risk of Receiving Health Care Disparities
Microaggressions in Science and Medicine	Assessment: https://implicit.harvard.edu/implicit/selectatest.html—Take the Race IAT Considering Microaggressions in Science https://www.ncbi.nlm.nih.gov/pmc/articles/PMC6007773/ The one who walks away from Omelas https://sites.asiasociety.org/asia21summit/wp‐content/uploads/2011/02/3.‐Le‐Guin‐Ursula‐The‐Ones‐Who‐Walk‐Away‐From‐Omelas.pdf Chika Stacey Oriuwa “In My White Coat, I’m More Black than Ever” https://www.flare.com/identity/black‐physicians‐in‐canada/

One week prior to each presentation, student presenters finalized a list of literature and media resources and sent out required readings to all journal club participants. The readings were chosen from academic journals, local and national news sources, as well as educational videos. There were no strict expectations for presentation content and resource lists, allowing each group to have creative freedom in choosing what and how to present so long as it followed our community guidelines to support respectful and thoughtful discussion. The freedom to individually design each presentation was believed to increase the level of presenter engagement and commitment to presentation topics. During the presentations, each group was responsible for teaching topic content, moderating group discussions, and fielding questions.

One of the key components of the journal club was creating discussion questions related to the required readings and key learning objectives for each topic. These questions were proposed to the group during the presentation and were discussed at length. Some examples of discussion questions included: “How might social and biological interpretations of race be used in basic research?”, “How does environmental racism factor in health disparities?”, “What surprised you about your Implicit Association Test results?”, and “What is different about the current conversations surrounding race, policing, and protests, compared to those at the time of Ferguson?” Discussion questions were chosen by student presenters and they were formed to be open‐ended and evoke open conversation to dive deeper into the topics. They also served to connect the topics to our future profession as physician‐scientists.

Following each presentation, resources and presentation slides were uploaded to a centralized folder on Dropbox so that the information could be accessed at a later time. All journal club meetings and presentations were held over Zoom to remain socially distanced. At the final journal club meeting, a post‐journal club survey was sent out to the group to determine overall satisfaction with the journal club and to assess comfort level and awareness of topics discussed throughout the summer. The survey was also used to guide further journal club programming to be continued throughout the school year.

## 6 OUTCOMES

### 6.1 Participation and learning outcomes

Twenty‐one students were invited to take part in the journal club. Participation in the journal club was optional. Out of the class of 21 students, 4 chose not to attend. The remaining 17 students attended at least one meeting over the course of the summer. Weekly attendance varied between 11 and 15 students per session, although a formal attendance was never taken. The demographic composition of the attendees consisted of 44.4% White students, 38.9% Asian students, and 22.2% Hispanic or Latino students based on a self‐reported survey completed by the participating students. There were no participants from the following demographics: Black or African American, Native Hawaiian or Pacific Islander, American Indian or Alaska Native. Since 38.9% of our participants were temporary visa holders, and would not be considered as an underrepresented minority (URM) by the National Institutes of Health guidelines, we analyzed the demographic composition based only on race and ethnicity and not based on citizenship status. Overall, 22.2% of our participants came from underrepresented backgrounds as defined by the National Institutes of Health racial and ethnic categories.^22^

Surveys were administered through Google Forms both before and after our 5‐week curriculum. Questions were aimed at understanding the personal perspective of cultural competency, perceived understanding of racism, comfort discussing race, perceived understanding of systemic racism, perceived knowledge of the history of St. Louis with regards to racism, perceived understanding of microaggressions, degree of feeling empowered to act on eliminating racism, familiarity with Black pioneers in science and medicine, and perceived knowledge of how to deal with microaggressions. There was no formal assessment to test true knowledge of each subject, so all measures are indicative of each students’ perception of their mastery of the material. Fifteen students responded to both surveys. According to the pre‐survey results, 11 incoming students (73.3%) had previously learned about racism in an academic setting and 4 had not.

One of our main goals in hosting a journal club was to discuss difficult topics, such as racism in medicine, in a safe, open environment, with the hope that this would facilitate collaborative learning. Based on the survey data (Table 2), there was an increase in students’ perceived understanding of racism after the journal club. Some other important improvements in self‐perception were increases in comfort talking about race, understanding of systemic racism, awareness of the history of race in St. Louis, and knowledge of how to respond to microaggressions. These results demonstrate that our 5‐week curriculum, utilizing an empowered leadership approach, positively affected the participants’ perception of key topics and introduced students to actionable items to implement going forward. In addition, the curriculum achieved a major goal of introducing students to the St. Louis region and the impact of race on its history, orienting students to their role as potential advocates for the surrounding community.

**TABLE 2:** Self‐reported survey results from Journal Club Participants

Survey measure	Mean student rating		*P*‐value
	Pre‐Journal Club	Post‐Journal Club	
Understanding racism	3.33	4.13	0.0038*
Comfort Talking about Race	3.4	4.13	0.0077*
Understanding Systemic Racism	3	3.93	0.0073*
History of Race in St. Louis	1.86	3.93	6.55 × 10^−5^*
Understanding Microaggressions	3.133	3.86	0.061
Feeling Empowered to Act	3	3.67	0.067
Familiarity with Black Pioneers	2	2.6	0.096
Dealing with Microaggressions	2.73	3.6	0.00149*

Pre‐ and post‐survey results to assess changes in the perceived understanding of topics addressed in the journal club. Students rated their level of perceived understanding on a scale from 1 to 5 where 1 is the lowest level and 5 is the highest level of understanding with the subject or comfort with the action. Data were analyzed using an unpaired *T* test. Significant changes (*P* < 0.05) in understanding are marked with an asterisk (*).

### 6.2 Lessons Learned

During the period in which we worked to design the curriculum and identify topics of interest, it became abundantly clear that participants had a diverse array of perspectives on which issues were important for us to learn about as a cohort of physician‐scientists in training. Two approaches that contributed to the efficacy of the curriculum's execution were allowing students’ self‐identification of topics of interest and disseminating teaching responsibility uniformly throughout our class. Both of these decisions in parallel gave students a voice in the direction and nature of the teaching materials presented, and additionally set expectations to produce high‐quality lecture and discussion materials for our classmates. This utilized students’ varying familiarities with preparation materials across a wide range of topics (history, sociology, clinical medicine, basic science) and sources (books, academic journals of various disciplines, news sources, videos) and produced a curriculum that was more interdisciplinary than could have been conceived otherwise. Doing so also capitalized on the respect fostered by our leadership structure by giving students the shared experience of facilitating a session. This ultimately increased engagement with the material and produced consistent attendance and participation across the cohort.

The use of defined discussion questions presented to the group during each session allowed students to interact and get to know one another, and actively engage with the week's material. Using discussion questions interspersed throughout the presentation, we were able to stimulate conversation regarding the material at hand; the questions not only gave students a prompt to which they could respond, but also a starting point to share their own thoughts and solicit the perspective of others in the group. Questions aimed towards assessing pre‐existing knowledge of the concepts presented also allowed facilitators and other students to understand the prior perspective of their peers and catalyzed the collective building of definitions. The community guidelines helped ensure that students remained respectful and mindful of others, creating a safe space that made students more comfortable and willing to share thoughts and experiences, especially when discussion questions solicited personal experiences. While it was understood that students need only answer if they felt comfortable sharing, many students were willing to share their personal experiences with concepts such as microaggressions.

### 6.3 Alignment with new Washington University Gateway Curriculum

Washington University School of Medicine underwent a curriculum renewal this academic year. The new curriculum places an increased emphasis on longitudinal community engagement, education of students on social and structural determinants of health, and improved understanding of the ways that inequities manifest in healthcare systems. The Gateway Curriculum topics strongly aligned with the self‐identified topics chosen by the MSTP journal club participants. Consequently, our journal club discussions prepared us with the background and familiarity to engage with these concepts throughout our medical education.

The Gateway Curriculum covered some of the topics previously discussed in our summer journal club, such as racism, ethics, and social justice in medicine, facilitating additional reinforcement and depth of knowledge. However, with our MSTP journal club, we were able to have intimate and open conversations amongst a smaller group of students with fewer time constraints as compared to the large lecture settings and substantially shorter discussions amongst students within the Gateway Curriculum. A second exposure to concepts such as microaggressions and bias, shared between the journal club's syllabus and the WUSM Gateway Curriculum, has allowed us to think about these topics not only through a medical lens, but also through a scientific one. Further, our journal club curriculum had a more in‐depth coverage of topics surrounding race in science. A deeper understanding of racial inequities in academia and science is especially useful to our MSTP class given our trajectory as physician‐scientists. We placed a large emphasis on learning how to conduct scientific research in an equitable, inclusive, and just way. We feel that longitudinal exposure to these concepts throughout our MSTP training will make our anti‐racism and social justice work more effective, impactful, and central to our careers one day as physician‐scientists.

### 6.4 Community building outcomes

At a time when health, safety, and social connection were dramatically impacted by the global pandemic, our journal club created a unique opportunity to socially connect with future classmates over topics that are critical to effectively addressing health and safety in our medical and scientific communities. After three planning meetings and five journal club sessions, participants felt comfortable contributing to a diverse array of discussions within our MSTP cohort. It was evident that the journal club dramatically enhanced class bonding, despite all encounters being virtual. The ability to establish class bonds through Zoom encounters was something both we as students and our administration were pleasantly surprised by. Not only did students get to know one another better through rich, and oftentimes personal, discussion, but presenter groups also bonded over creating a portion of the collective syllabus and coordinating a presentation of the material.

The development of the journal club also coincided with the unexpected, and later retracted, Immigration and Customs Enforcement directive to deport F1 and M1 visa students participating in 100% online curricula in the coming semester.^23^ The journal club helped to support the 38.9% of our MSTP class that are F1 Student Visa. First, it gave us a space to share our fears surrounding the volatility of visa statuses in America, and the immediate impact these actions would have on diversity and inclusion in medicine and science. We also planned to use our journal club as a ready‐to‐go “face‐to‐face” class in the case that the ICE ruling was implemented or future governmental rulings threatened our visa statuses.^24^ Thankfully, this was not needed, but the support from our classmates contributed to the feeling of community amongst our class, even before our official start at WUSM.

## 7 CHALLENGES

There were challenges associated with executing an entirely student‐directed curriculum as the entering MSTP class during a global pandemic. One of these challenges is related to the empowered leadership structure that we adopted. While it was a strength that all participants had the opportunity to educate the class on a specific topic, none of us had expertise in the topics covered. This lack of expertise made it difficult to bring in opposing viewpoints, where different academic branches had different stances on a topic. For example, some academic sociology circles oppose the idea that there is any shared biological basis within a racial category, while there are trends in academic medicine which rely on race‐specific pharmacological or physiological differences to explain racial differences in outcomes and metrics.^25^, ^26^ In these cases, the absence of a designated source of expertise and authority made it difficult to bridge conflicting schools of thought and occasionally left us without satisfying conclusions to discussions. Therefore, we hope that occasionally bringing in experts on specific topics we discuss could aid in our longitudinal understanding and implementation of journal club topics.

Another challenge we faced was taking specific actions based on and inspired by our curriculum. While COVID‐19 has posed an unprecedented obstacle in utilizing our knowledge, the desire to funnel our efforts to supporting community‐initiated programs with an emphasis on responding to the needs of the community has impelled us to pause and thoroughly research how we can help in a positive way. An avenue through which we may take action in the near future is through community engagement opportunities introduced by the facilitators of the WUSM Gateway Curriculum. These community engagement opportunities have been vetted by the facilitators as ways to collaborate with the community to bring about change and improve well‐being and health, while deferring to the expertise of organizations and activists local to St. Louis. Further, these opportunities are in response to conversations with community leaders to ensure that Washington University's actions are specifically addressing the needs that the community has voiced and intervening to the degree the community desires.

## 8 LIMITATIONS

While the journal club was designed to be voluntary, this limited the strength of our data analysis. A variable number of students joined our online discussion every week and the survey respondents from the beginning and end surveys were not paired. This variability in participants meant that statistics had to be analyzed using an unpaired student *t*‐test approach. In addition, the sample size was low because this journal club was proposed to our 21 person MSTP class, which resulted in low power in our analysis. The creation of the survey to measure the learning of self‐perceived growth in understanding health disparities and racism was difficult to quantify and does not necessarily correlate with objective learning. Eleven students already had a self‐perceived understanding of racism before attending the journal club, which made it harder to determine the impact of some of our sessions. An improved way to measure learning across this small cohort will be to create more data points as our journal club progresses and continue to implement surveys to understand the benefits of this type of engagement. For example, we could implement more objective measures of learning based on material taught and discussed during each journal club meeting over longitudinal timepoints, similar to previous studies focusing on assessing the effects of an indigenous health curriculum through knowledge recall or measures of ethnocultural empathy.^27^ This longitudinal approach of understanding the impact that journal club discussion has will also benefit learners in creating an optimal journal club that continues to meet the unmet needs of other academic activities in addressing racism. Despite these limitations, the knowledge amongst our class contributed to the student‐led presentations and in‐depth discussions. In addition, the small sample size did not detract from one of the main goals of this program, which was to provide an example curricula that could be emulated by MSTPs across the country, many of which are less than 25 students per class.

Another important limitation of our journal club was the lack of specific underrepresented groups, including Black and Indigenous students. In total, 22.2% of the participants of our journal club fit the NIH criteria of URM, contributing to the varied perspectives in our presentations and discussions. However, since anti‐racism was a major value behind the implementation of the journal club, the lack of Black students in our group was very apparent, which we discussed during our sessions. Presenters made sure to reference Black authors and experts, including them in mandatory and optional resources. However, this does not replace the voices of Black students. We have expressed this dearth of representation to our MSTP directors and we have been in communication with upper year Black students in the WUSM MSTP. We have voiced our support of their efforts for increased diversity and inclusion in the program, we have solicited input on topics and resources for our future journal clubs, and we have opened the opportunity for them to join our journal club to share their experiences and perspectives. The MSTP office has responded to the concerns of our classmates by hiring a Diversity, Equity, and Inclusion administrator and focusing on undergraduate diversity recruitment programs. Further, the MSTP students and administration gathered at a virtual Town Hall meeting to discuss and begin the creation of a student‐run “Diversity Sub‐Committee" within the WUSM MSTP. This sub‐committee will work on recruitment programs, education, and improvement of MSTP culture around racism.

Our exact journal club structure may be difficult to replicate across other MSTP programs since the topics of discussion were chosen to be specific and connected to the St. Louis community. We believe that this approach of planning, implementing, and reflecting is still very valuable for other cohorts that are interested in addressing health disparities in science and medicine. This curriculum was created in the time of a global pandemic and was completely virtual, so this model may look different for future years where a shift back to in‐person learning may occur. In addition, there was no centralized leadership for this journal club. This made the presentations, content, and engagement completely voluntary. The main incentive to participate was the willingness to invest in learning about racism and health disparities in healthcare and research. Students that participated in this journal club felt empowered to participate in these discussions because there was a safe space and environment, which our MSTP directors contributed to. If other program directors wish to encourage students to start something similar, it is important to create a space where discussions about addressing racism are brought to the fore as a commitment of the program.

## 9 FUTURE DIRECTIONS

As MSTP students, we are training to play integral leadership roles in science, medicine, and the translational interface between the two fields. Given our future leadership roles, we will have the platform and authority to advocate for change and move our fields in directions that actively oppose racism and injustice. This is why it is especially important for us to be aware of different issues surrounding social injustice and to be comfortable discussing these issues and the different points of view around them.

Additionally, as MSTP students, we will be residing in St. Louis for at least 8 years, which provides us the opportunity to be involved with the community in a longitudinal and significant manner. Long‐term community engagement would allow us to build trust and deeper connections with community leaders, making community members more comfortable and more receptive to our involvement in addressing their needs. To be able to truly make the most out of our time spent engaging with the community, we must be aware of social and structural issues that individuals face. The MSTP journal club provides the framework for us to learn, discuss, and repeatedly think about these issues. Repeated exposure and application of these frameworks will allow us to practice trauma‐informed care, community engagement, and ethical scientific research.

As a cohort, we plan to continue this journal club throughout our tenure as MSTP students at WUSM. So far, we have had several organizational meetings and integrated feedback from the summer surveys in order to continue meeting as a journal club on a monthly basis. We plan to revisit the community guidelines each semester to ensure it still aligns with our goals. During the academic calendar year, we have continued to hold journal club meetings once per month for two hours. Discussions continue to be student‐lead and focus on different topics each month. Students are able to sign up to present articles, media, books, or other materials relevant to the topic for that month. We also plan on holding an action item meeting once per month, where we will discuss ways in which we can actively contribute to the St. Louis community. With the leadership skills we have and will continue to develop, we hope to make a long‐lasting impact in the St. Louis community.

The inaugural MSTP journal club participants have two long‐term goals: to officially incorporate the “Social Justice in Medicine and Science Journal Club” into the WUSM MSTP curriculum and to facilitate the implementation of curricula like this in other MD‐PhD programs across the country. The long‐term effects of this journal club will positively benefit physician‐scientist communities at all levels of training. The Tri‐I MD‐PhD class also plans to continue their journal club in future years and has been working with Tri‐I program leaders to incorporate the journal club into the formal summer curriculum for future Tri‐I classes. Similar initiatives of social justice and anti‐racism journal clubs are currently implemented at only a few other institutions like the University of Cincinnati, University of California San Diego, and University of California Irvine, all of which appear to be mostly faculty‐led.^28^, ^29^, ^30^ This highlights the need for additional institutions to participate in these initiatives. To motivate other institutions to adopt similar practices, we plan to make both our materials and curriculum structures available on MedEd Portal in the coming year. We will also act as a point of contact for those institutions and students who hope to implement similar longitudinal programs for their MD‐PhD cohorts.

## 10 CONCLUSIONS

The development and implementation of a social justice and anti‐racism journal club for MSTP students at Washington University School of Medicine was motivated by an urgency for action during an extraordinary global pandemic which has reinvigorated the ongoing movement for racial justice, along with the police killings of Ahmaud Arbery, Breonna Taylor, George Floyd, and countless others. For us first year MD‐PhD students, the journal club provided us with not only an opportunity to learn about the ever‐present structural inequalities built into medicine and academia, but also a safe space where we could reflect on our current and future roles within these pre‐existing frameworks. In particular, the flexible “shared leadership” structure of the journal club was critical to our success in developing a well‐rounded curriculum and promoting respectful dialogue among students. Looking forward, we recognize that this journal club ultimately represents a small step in a lifelong journey to learn about and ultimately combat systemic racism in our future careers as physician‐scientists. However, we are committed to the continued evolution of this journal club as a vehicle for positive impact in our local and national communities during our longitudinal training as MD‐PhD students. We hope that our experiences will serve as a much‐needed prototype for student‐led social justice initiatives in medical education.
